# Book Reviews

**Published:** 1822-05

**Authors:** 


### CRITICAL ANALYSIS

OF

### ENGLISH AND FOREIGN LITERATURE

RELATIVE TO THE VARIOUS BRANCHES OF

### Medical Science.

Quæ laudanda forent, et quæ culpanda, vicissim

Illa, prius, cretâ; mox hæc, carbone, *notamus*.—PERSIUS.

### DIVISION I.

### ENGLISH.

ART. I.—*Remarks on Cutaneous Diseases.* By J. H. WILKINSON. 8vo. pp. 87. J. and A. Arch, London. 1821.

THE author of the above pamphlet has been some time known to the public, as an extensive practitioner in that branch of the science of medicine to which it relates; and we have, therefore, expected his publication with some anxiety since its announcement to the world, not doubting but that it would contain the results of extensive observation and inquiry, and such practical deductions as could be received, and acted on, by the profession, with unlimited confidence. Whether the obstacles to the attainment of satisfactory conclusions be, however, of a more insurmountable character in cutaneous than in other diseases, and, consequently, our expectations on this point have been too sanguine, we shall not pretend to determine: certain it is, that, if the number, importance, and originality of facts, are to stamp the value of the publication, we shall have no difficulty in enabling our readers to form a proper estimate of it: if it determines the extent of attention we are to exact from them to our analysis, their task, as well as our own, will be very speedily performed.

The most discouraging and prominent feature in the publications of authors of the present day on Cutaneous Diseases, is an endless diversity and opposition of opinion in almost every thing which regards their pathology and treatment:—discouraging, because it justifies the inference that but little is with certainty known; and, as an instance of the prominence which such feature has assumed, we shall direct the attention of our readers to the works of ALIBERT, (who has been pronounced, by a contemporary, the greatest dermologist of the present day,) and submit the following extract from the work before us.

"From the perusal of Mons. Alibert's inflated fustian, in his *Description des Maladies de la Peau*, I rise (as I think every sober man must rise,) puzzled and disgusted. Surrounded with greater advantages than any other surgeon in Europe, and with Dr. Willan's work open before him, he has neglected that learned author's beautiful arrangement; and has contrived, by his perplexing pathology, and by his inefficient and contradicting remedies, to render this department of science more confused and incomprehensible than he found it."* (p. 74.)

Mr. Wilkinson commences with the expression of his opinion,

"That the secretion upon the surface of the true skin, and its power, when altered from its natural state, of producing all the different eruptions, has never been properly or sufficiently considered; nor yet how dependent their varieties are upon the different states or conditions of the skin, upon which such disordered secretion acts.

"The prevailing opinion, that different eruptions are produced by different diseased secretions, appears to me to be incorrect; since, I believe, that the diseased secretion is always essentially the same, and that the varieties of the eruptions are produced solely by the conformation and constitution of the subject." (p. 1.)

We shall have occasion to remark further on the above opinion as we proceed. Thinking as we do, however, somewhat differently on this point from our author, and that it is this very propensity to generalization, so strongly exemplified in the above passage, which has hitherto kept us in the back-ground in the management of cutaneous diseases, we are not disposed (and we think the majority of our readers will agree with us,) to accept the following as a very forcible illustration of the correctness of his position.

"We continually observe, that the same cause produces very different effects in different individuals; that the same medicine vomits one person, purges another, and excites diaphoresis in a third; that the cold damp air acting upon two people, at the same moment, in the same situation, produces, in one a pleurisy, in the other typhous fever. These circumstances are familiar to us all; and we easily explain them by considering the different habits, and the different states of the system, of those who are at the same moment exposed to the same causes." (p. 3.)

We may be permitted to observe, that the fluid secretion of itch, of vaccinia, variola, &c. produces a perfectly corresponding diseased secretion in every state of system; and, so far as our own observation goes, the same remark may be made of the porrigo scutulata and favosa: and, though we are ready to

---

* We would have difference of opinion expressed in such language as should lead to temperate and instructive discussion; for, only so expressed, can it be likely to encourage that feeling towards each other, on which the true interests of the science, and our own estimation in society, must so materially depend. Sentences like the above are beneath the notice of legitimate criticism, and incompatible with respectable authorship.

admit that the state of system, or degree of irritability of the skin, may determine its susceptibility of the action of the matter applied to it, we must doubt the possibility of the matter of one producing the other disease, as the consequence of any state of system previously existing; and, if our readers agree with us, they will at once see that the diseased secretion is *not* always essentially the same.

"It is not necessary at present to state the general causes of such diseased secretion; but, whatever it may be, (for instance, a long-continued interruption, or irregular action, of the digestive powers,) I have no doubt that such cause will produce, on the skins of different persons, the different diseases mentioned;* which may, therefore, be cured by the same remedies." (p. 3.)

Of this passage we will merely observe, that its incorrectness may be inferred from the practical fact, that the most successful remedies in the greater part would be decidedly improper in the remainder. In fact, we see nothing in it but a more blind adoption of the digestive-organ pathology, than that which the author has so severely noticed in another part of his work. The principal observations he has submitted in support of his theory, we have no hesitation in pronouncing to be utterly destitute of weight; for they merely show that different degrees of susceptibility of the power of infectious matter exists in the cutis of different subjects. Thus, for instance, he observes, "even the itch, which is generally very quickly communicated, will not affect some skins, though exposed to it for months; and it will exhibit itself on some as a vesicular, in others as a pustular, disease." (p. 4.)

Our author remarks, that the *skin of the scalp seems to differ, in its nature or texture, from the skin of other parts of the body;* and a child, affected with any variety of tinea capitis, may, by communication with his fingers, produce an eruption on other parts of his body; but such eruption will seldom bear much resemblance, and frequently none, to the tinea which produced it.

In a little essay, which passed our critical tribunal some months since, we notice the same observations followed by an apparently satisfactory explanation of the former fact, and an accurate description of the cutaneous affection alluded to, as produced by the application of matter from the diseased scalp to the skin of other parts.† We are, notwithstanding, far from suspecting Mr. W. of any thing like plagiarism; as there are

---

* The whole of the three orders of Squamæ, Pustulæ, and Vesiculæ; comprising lepra, psoriasis, pytyriasis, ichthyosis, impetigo, porrigo, ecthyma, variola, scabius, varicella, vaccinia, herpes, miliaria, eczema, and ephtha!

† *A Practical Essay on Porrigo.* By Samuel Plumbe, Esq. Member of the Royal College of Surgeons, &c. &c. &c.

evident proofs, elsewhere, that the work in question has not passed under his notice.

After some remarks, *en passant*, on the work of Dr. Willan, and the fondness of this author, and others, for Latin and Greek quotations, "which, if not intended to exhibit the erudition of the author, must be intended to enlarge the work, and to increase the price of it," we are led to the consideration of the benefits of volatile alkali in the malignant scarlet-fever; and think that Mr. W., in thus bringing forward this medicine to the more extensive notice of the profession in the above disease, deserves our warmest thanks. In that low and exhausted state of the system, which often supervenes in a few hours after the appearance of the eruption, both in this disease and measles, and sometimes terminates in a train of alarming symptoms, it is, doubtless, an invaluable remedy. The clearest indication in this state of matters, must be to rouse the energies of the nervous system, and give, by temporary excitement, such vigour of action to the heart and arteries as will enable them to keep up that regular determination to the skin, on which the safety of the patient may be pronounced to depend.

In the above remark we are neither assenting to, or dissenting from, the opinion that copious perspiration is never to be expected during the continuance of the eruption of scarlatina. It is certain that common diaphoretics do not usually produce such an effect; but we have reason to know that the carbonate of ammonia, liberally used, does occasionally possess this desirable power. In fact, we have a patient now under our eye, who, but yesterday, had as dark a coloured scarlet skin as is often seen, and perfectly free from moisture; sloughy sores on the tonsils, surrounded with a dark purplish-red areola of inflammation; great prostration of strength, and a state of the circulation which precluded a possibility of feeling the pulse at the wrist. She took the ammonia in ten-grain doses, repeated every four hours; and, at the period when she was visited today, (about half-past eleven, A.M.) the skin was covered with perspiration; her general sensations wonderfully improved, and her pulse, though hurried, perfectly distinct and soft:—a description of change, in so short a time, which it has never been our lot to witness under other plans of treatment.

Mr. W.'s opinion of the inferiority of other medicines and plans of treatment to the use of the ammonia, may be collected from the following extracts:—

"In 1803, I attended several cases of the scarlatina maligna, with Dr. Willan and the late Dr. Hamilton. It is well known that the disease raged most fatally during that period, and we lost four of our patients out of five in one family. Never were men more puzzled to know what remedies to adopt. All which Dr. Willan has recommended in his publication were employed. Emetics, purgatives, calomel, and antimony; many other diaphoretics; opium, wine, and acids; bark, blisters, decoct. contrayerva, with oxymel of squills; application of cold water, gargles of different descriptions, fumigations, &c.; all without the least good effect,—all without making the least sensible impression upon the disease in any of its stages.

"About this time, Dr. Peart published his "Practical Information on the Malignant Scarlet-fever and Sore Throat," in which he describes the wonderful effects of the subcarbonate of ammonia, and considers it to be endowed with a specific power over that disease. Like other practitioners, he was continually lamenting the loss of his patients by that dreadful malady, till he employed the ammonia; and, from that moment, he did not lose one patient out of nearly three hundred." (p. 13.)

Having been thus led to the trial of the medicine, Mr. W. informs us that he has "not only never lost a patient in this disease, but has never had a case of the kind that has even appeared dangerous, or given him a moment's anxiety."

The importance of this medicine, among the class which has received the appellation of diffusible stimuli, is now pretty generally understood. Its almost resuscitative powers in many cases of exhausted nervous energy, must make it a desideratum in instances where the feelings of the physician go hand-in-hand with those of the friends of his patient; where despair may be shut out, and the enlivening hope of recovery substituted in its stead, if life (and there are many such cases occur,) can be, by art, supported but for a few hours.

In the treatment of obstinate cases of the lichen circumscriptus and agrius of Dr. Willan, independent of a regular plan of mercurial alteratives, Mr. W. has found the application of aromatic vinegar* to the part, of great utility in allaying the irritation and itching of the skin, and preventing the formation of troublesome and painful fissures. He has not found any bad effects from the removal of the eruption in this way, but is in the habit of employing the ammonia internally at the same time; exhibiting it in doses of five or six grains every four hours.

"I dilute a little aromatic vinegar with one-third of water; and, with a piece of lint wrapped round a probe, and moistened with this acid, I slightly touch the parts which are most affected with the itching, heat, and painful tingling. This I do every day, or every other day; and, in the mean time, I prescribe the following lotion:

* The composition of this medicine seems to be extremely doubtful. As prepared in the shops, it is probably materially different from the formulæ given in the Dispensatories. This, however, is not of much importance, since its properties are merely those of a common vesicatory or caustic, according to the manner in which it is applied.

℞. Ammon. Subcarb.

Plumb. Superacet. ā ℈j.

Aq. Rosæ, ℥iv. M. f.

and direct that the same parts be moistened with it, whenever troubled with the above-mentioned sensations." (p. 25.)

The like flattering success has, in the practice of our author, attended the application of the aromatic vinegar in distressing cases of prurigo; and he has recorded one instance, the details of which we shall lay before our readers.

"About five years ago, I was requested to see an elderly gentleman, living four miles from town, who was afflicted with an eruption, which, I was informed, had nearly disordered his senses. When I entered his chamber, he was sitting up in bed with his shirt off; and a most piteous spectacle he presented: his body was besmeared with blood; he was, at the time I entered, tearing his skin with a comb; and he informed me that, for some months, he had employed his nails, but that lately they had been incapable of producing a smarting sufficient to overpower the dreadful itching, and therefore he was obliged to have recourse to the comb, which he employed in that manner two or three times in the course of every night. I found that he had been subject to slight attacks of this complaint for years, at different periods; and that it had been, more than once, treated as scabies; that he had been possessed of great property, but that it had lately been considerably reduced, by means, to his mind, the most distressing; and there is no doubt but that this disease, which is always exasperated by every painful affection of the mind, was rendered so peculiarly afflicting by such circumstance.

"He had taken, during the last three months, from different medical gentlemen, sulphur, sodæ carbon., decoct. sarsæ. comp., cinchona, many different and strong purgatives, and the mineral acids. He had used, externally, warm baths, with and without salt, liq. ammon. acet., lot. saturn., ung. hydr. rubri, ung. precip. alb., et aq. calcis cum calomel. None of these had been capable of abating the intolerable itching of the skin, sufficiently to procure him two hours' sleep during any one night. He was considerably reduced both in flesh and strength; and his spirits were so much exhausted, that, for the last fortnight, he had thought it necessary to drink a bottle of Madeira every day.

"I dipped some lint in the aromatic vinegar *undiluted,* and touched most of the prominent papulæ with it, bleeding as they were from the laceration of the comb, till the sense of smarting was as much as he could bear: though at the height, he declared it preferable to the itching. I then sent him the following ointment:

℞. Sulph. Sublim.

Picis Liquidæ,

Axung. Porcin. ā. ℔ss.

Terræ Cretōs. iv.

Hydrosulph. Ammon. ʒij. M. ft. unguent.

"I desired him to apply this ointment liberally over the whole extent of the eruption; to renew it every day, and wash it off every other day. I gave him four grains of pil. plummer every night, and five drops of sol. arsenic three times a-day. Wine, salt provisions, shell-fish, and every stimulating article of food, were forbidden.

"During the second night after these applications, he slept above four hours; and the itching was considerably abated during the day. In three days, the acid was applied a second time; and afterwards a solution of argent. nitr. every third or fourth day, previous to the ointment.

"In less than three weeks, the patient was very comfortable; the eruption having nearly disappeared, and the itching being entirely removed.

"The pills and solution were continued for three weeks longer; during which time a lotion of hydr. oxym. et spir. vin. rectif. was applied two or three times a-day, instead of the ointment." (p. 27.)

'We have arrived at this part of our analysis with no small degree of pleasure; for, in addition to the satisfactory feeling which the signal success of a new remedy in an hitherto unmanageable complaint, (an instance of which is presented in the case before us,) is calculated to excite, we find our author asserting that, "principally upon this plan, and with these remedies, he has for many years succeeded in curing, with comparative ease, the following diseases: viz. Prurigo, lepra, psoriasis, pityriasis, impetigo, porrigo, scabies, herpes, sycosis, and all their varieties, except one variety of herpes, the *zoster*." We sincerely hope that the flattering prospects thus held out to us and our professional brethren, will be realized by the results of our own practice: but, accustomed as we have been to disappointments of this description, we shall, in the present instance, be prudent enough not to be sanguine, and endeavour to believe that it is possible that, in other hands than our author's, the aromatic vinegar may prove as destitute of its reputed virtues as the celebrated Theives' vinegar of old. We are not old in the profession; but we have outlived the reputation of many medicines and medicinal applications, affording, by their known properties, better prospects of accomplishing our objects in their use than that under consideration.

The part of the work relating exclusively to the varieties of porrigo occupies about forty pages; and here, in conformity with the author's proposition as to the original identity of all cutaneous affections, we find him boldly asserting, that any one variety of this disease is capable of producing, by infection, the whole of the others. He appears, however, to anticipate a little incredulity on the part of his readers on this point; for who, he asks, "*without experience*, could believe that the porrigo favosa and decalvans are the same malady?" We firmly believe that

Willan himself was far from satisfied of the propriety of placing the latter affection in the class Porrigo; for he had only seen one or two cases of it, "where the P. scutulata had spread through a large school or seminary;" and his opportunities of observation must have been pretty extensive, for, in the course of a short period, children were brought to him from 124 different seminaries, affected with the latter disease.

From Mr. Wilkinson's own account, the porrigo decalvans never exhibits the slightest vestige of pustulation, or secretion of moisture of any other character. In what point, then, does it answer to the definition of porrigo, which is "a contagious disease without fever, exhibiting an eruption of the pustules termed achores and favi?" Our own conviction is, that the phenomena of porrigo decalvans are entirely peculiar; that they are produced by the cessation of action of the organic structure secreting the hair, and are independent of the slightest disease of the cutis of the part.

We pass over the consideration of the P. larvalis, furfurans, lupinosa, and favosa, not being able to collect, either from their description or treatment, any new fact of importance; unless, indeed, the statement, in opposition to the opinion of Dr. Willan, that the P. furfurans is more common in children than adults, can be so considered. An error in judgment of "some of the most eminent practitioners of the present day," is noticed in the discussion on the former of these, the porrigo larvalis. According to Mr. W. this affection has been considered by those to whom he alludes, as depending on *poorness of blood*, and is, therefore, very much maltreated and aggravated: but we are not disposed to believe that this egregious error is so commonly committed at the present day as he imagines; or that medical men are so blind to the obvious connexion it generally has with an opposite state of system, as to furnish, very frequently, such cases as that which he has detailed for animadversion.

As regards the remedies, no distinction has been thought necessary for the different species of porrigo, except that the solution of argent. nitras is applied more liberally to the scutulata and lupinosa than to the others; the whole of the medicinal treatment, independent of occasional alteratives, consisting of the application of this solution every other day to the diseased scalp, and the use of the unguent. pius et sulphuris in the intermediate time.

"Thus, for all the varieties of porrigo, as for all the varieties of squamous eruptions, I depend upon the same remedies, applied in the same manner, and upon the same internal medicines; and very rarely do I meet with a case which requires any thing more than a proper regulation of such remedies." (p. 56.)

The last division of the work, headed "Impetigo, &c." is occupied by the statement that this disease, the grocer's and bricklayer's itch, the sycosis menti, and scabies, are treated successfully with the remedies which have been already mentioned; and the latter only requires sulphur internally; that, in a case of lupus, the trial has afforded results equally promising: the diluted acet. arom. being first applied to the diseased surface, and followed by the use of the tar and sulphur ointment; the arsenical drops and alterative pill not being forgotten.

"To the varieties of acne I apply the aromatic acid twice a-week, and a solution of hydr. muriat. in spir. vin. twice a-day; and, when I employ an ointment, as nothing offensive can be applied to the face, in such cases I use the ung. zinci, the alterative pills and drops are given as before mentioned." (p. 83.)

We have now given a pretty copious analysis of a work of eighty-seven pages, on a subject affording an ample field for practical inquiry on a class of diseases, to which a very small portion of the medical talent of Europe has been hitherto directed; and, when we consider the great importance of the subject, and the opportunities which our author has had of prosecuting a system of industrious research, we must confess that we rise with a feeling of considerable disappointment from the perusal of his work. So far as our own experience goes, (and we have not suffered this branch of pathology to pass unnoticed,) our only hope of arriving at a system of practice in cutaneous disease, equally successful with our management of others, depends upon a minute attention to the state of vessels on the diseased surface, and the relative connexion such state may appear to have with the hereditary, or temporary, conditions of the system. We have not, till lately, been placed in a situation favourable to the successful prosecution of inquiry as to this class of diseases; but a ray of hope has lately appeared on the horizon, in the shape of an institution expressly directed to this object in the metropolis; from the known talents of the medical officers of which, many and great improvements may be reasonably expected.

**Art. II.—*An Essay on the Symptoms and History of Diseases; considered chiefly in their Relation to Diagnosis.* By Marshall Hall, m.d. f.r.s.e. and formerly Senior President of the Royal Medical Society of Edinburgh. 8vo. pp. 130. Longman and Co. London, 1822.**

Dr. Hall seems fully alive to the magnitude and practical importance of symptomatology. His work on Diagnosis must be known to most of our readers, who will recognize, in the present essay, the first part of that performance. As a new edition, therefore, of an old work, we are not called upon to say much of the volume before us; but the subject on which it treats is of such moment, particularly to the practitioner who cannot yet boast of much experience, that we are willing to dilate upon it.

It stands recorded, as the opinion of the Father of medicine, that to know a disease is, in fact, to cure it. "Medicus sufficiens ad morbum cognoscendum, sufficiens est ad curandum." We are not prepared to go the whole extent of this proposition. Nothing is so common as to meet with medical men, endowed with great quickness of apprehension, exquisite judgment, and a high degree of the *tactus eruditus* in ascertaining the true nature of a disease, to which they will often apply the most absurd or inert remedies. We are acquainted with a very eminent physician, whose unerring *swiftness* in naming the disease, in cases of infantile complaints, is both unquestionable and unprecedented. Having followed, for nearly two years, his clinical visits, we can undertake to assert that his diagnosis has never been belied by necroscopical examinations, whenever an opportunity for the latter has presented itself. Yet, *cui bono* all this acumen,—this enviable gift of discrimination? if, after all, the remedial agents called to the assistance of the young sufferer are of the most inert class?

We are, however, perfectly ready to admit that the medical practitioner, who does not make semiology his particular study at the bed-side of his patients,—or who, during his medical education, has neglected this highly necessary branch of medicine,—will never be able to practise satisfactorily to himself, or beneficially to those who are entrusted to his charge.

Various are the works that have, at all times, been written on Diagnosis, Semiology, and Symptomatology. Every author has adopted, more or less, a new mode of arranging diagnostic signs from that of his predecessor; but, as yet, no arrangement has been proposed superior to that of Professor Chaussier. Dr. Hall has very judiciously adopted it, with very slight exceptions; although we are not aware that he has mentioned the name of that distinguished physician and writer in any part of his present work.

Professor Chaussier considers every symptom indicative of deviation from health, as susceptible of being classed under one of the following heads:

1. The countenance.
2. The attitude.
3. The skin.
4. The vital functions.
5. The sensorial functions.
6. The nutritive functions.
7. The generative functions.
8. Individual and local circumstances; including the consideration of the age and sex of the patient, its primitive constitution, stature, size, conformation of the body, regimen, manners and habits, passions, education, habitual occupation, &c. &c.

The first part of Dr. Hall's essay, which is exclusively consecrated to the enumeration of symptoms of diseases, is divided into eleven chapters; each of which, being designated by some general title, will be found to correspond to the French professor's subdivisional arrangements.

Chap. i. On the morbid appearances of the countenance.
— ii. On the morbid conditions of the attitude.
— iii. On the morbid appearances of the tongue.
— iv. On the morbid conditions of the general surface.
— v. On some morbid conditions of the general system.
— vi. On the morbid states of the function of the brain.
— vii. On the morbid affections of the function of respiration.
— viii. On the morbid affections of the circulation.
— ix. On the morbid affections of the functions of the alimentary canal.
— x. On the morbid affections of the functions of the urinary organs.
— xi. On the morbid changes in the functions of the uterine system.

We do not agree with the present author in thinking that the study of the symptoms and history of diseases has, hitherto, been treated in an imperfect manner. The number of works that have been written on the continent for the improvement of this branch of our art,—the establishment of clinical wards every where, except in the great metropolis of Britain, for the purpose of watching the permanent, as well as the transitory, physiognomy of disease,—the close and repeated examinations of candidates for the *laurea*, adopted at most universities, on questions of pure diagnosis,—sufficiently proclaim, how fully alive the profession has been, of late years, to the importance of diagnosis; and what consequent measures they have adopted to facilitate its study. It should also be stated, much to the credit of modern writers in physic, that the history of diseases, and the enumeration of their symptoms, are much better treated, now, than they have been at any period since the introduction of the greatest improvements in the healing art.

There are two French works on the value and nature of signs and symptoms in diseases of recent date, which, although not mentioned by Dr. Hall, we must recommend to the attention of our readers, as by far the best which the profession possesses on these matters; greatly superior, indeed, to the small volume we are reviewing. We mean those of Dr. DOUBLE and Dr. LANDRÉ-BEAUVAIS.

We however wish not, by any means, to under-rate the importance of Dr. Hall's little performance. Considered as an endeavour, on his part, to direct the attention of the junior branches of the profession in this country to the study of diagnosis, and as an attempt to supply them with a syllabus of that science in a concise and cheap form, we are glad to have it in our power to recommend it.

There is a suggestion in one of the pages of Dr. Hall's essay, which we consider of much importance.

"We still want an essay on the morbific effects of remedies:—1, when misapplied; 2, when even appropriately, but perhaps injudiciously, administered; and, 3, from idiosyncrasy. I may instance blood-letting, and purging, and opium, as productive of morbific effects of the most serious character, to which my attention has been particularly directed: it is needless to add to the list mercury, digitalis, cantharides, &c. with which every practitioner has learnt to associate certain morbid conditions of the system."

The second part of this essay is on the history of diseases, and consists of two chapters: the first treating of the principal points comprised in the history of diseases; the second giving a sketch of a few diagnostic and practical rules for dispensary practice.

We shall give an extract from the latter, merely as an introduction to a few remarks of our own, on the common mode of examining patients, and the necessity that there is for a physician to attend more closely, as well as systematically, to the investigation of the nature of a disease, when first called in to a patient, to the progress of the complaint, its variations and transitions, to the curative or morbid effects of the remedies employed, and to the impression caused on the mind of the patient by our mode of practice, and our delivered opinion.

"The first question to be asked of the patient is, how long he has been ill? The reply resolves the case into the class of acute or of chronic affections. The former are, principally, fevers, the acute dyspepsiæ, or acute inflammations; the latter are the chronic dyspepsiæ, the insidious organic diseases, or the insidious forms of inflammation,—especially of the brain, the pleura, and the peritonæum.

"Having thus ascertained the class of the disease, we must proceed, in the case of the acute, to investigate the individual nature of the case. In chronic affections, we may ask—

"In the second place, whether there be a material and progressive loss of flesh?

"The reply to this inquiry divides the cases into such as may subsist without influencing the nutrition, and such as gradually reduce the patient. The former cases are chiefly the chronic and protracted forms of the dyspepsiæ, or diseases of such organs as are not engaged in the process of assimilation. The latter are marasmus, phthisis, mesenteric disease, chronic inflammation of the peritonæum, and in general diseases of the organs of supply.

"A third inquiry is into the state of the pulse. Increased frequency of the pulse is the usual attendant on the insidious forms of organic disease; whilst it is not observed in the less serious cases of the chronic dyspepsiæ."

It has often been mentioned to us, as a cause of complaint in private practice, that certain physicians, whether from multiplied occupations or constitutional indifference, give but little of their time to the consideration of the case to which they are called; that some will not permit the patient to speak; while others scarcely ask the patient a single question. Few, if any, trouble themselves much about examining or inquiring into the nature of the excretions; and most of them grant but a few minutes to each case. If this be a real picture of private practice, then we have no hesitation in saying that the patient is defrauded of his fee-money,—his life is trifled with,—and the physician draws down discredit on himself and the profession. So many are the obstacles which naturally present themselves to a practitioner in forming a correct diagnosis of a disease, under even the most favourable circumstances, that, however skilful and experienced that practitioner may be, he needs the assistance of every minute particular detail, whether obtained through his own examination, or the voluntary exposition of the patient, in order to arrive at the truth. Else, how will he be able to give the disease its proper name, or apply to its removal the necessary remedies? What chance has he of forming a correct prognosis of its result, so as to satisfy the friends of the patient? It has come to our knowledge that, in a recent instance of death from uterine hemorrhage, in a lady of rank, the medical attendant had, but a few hours before, made light of the case, and assured the family of its happy termination, in opposition to the apothecary, who, having closely watched the noble sufferer, had prepared her husband for the inevitable catastrophe. Now, in such a case as this, the medical person to whom we allude had surely given himself too little time to consider the malady, and reflect on its probable termination. If he left the patient still labouring under profuse hemorrhage, (which we understand to have been the case,) how could he depend, with any certainty, on the disease terminating successfully? —Doubtlessly, it was not the treatment adopted on the occasion, (phlebotomy! in passive hemorrhage immediately after labour!) which could warrant him in assuring the friends that the patient was safe. In another case, well known to the profession, an elderly lady exhibited every symptom of irritative fever, from some affection of the stomach and bowels. The symptoms were immediately ascribed to dyspepsia; and the blue-pill, with drastic purgatives, was given. The previous history of the patient's life and former complaints had never been investigated by the attending physicians. After death, it was found that the small intestines were plugged up with conglomerated masses of magnesia!

On the whole, we think that the physician or surgeon, who, from any motive whatever, omits entering into an extensive inquiry of every particular, respecting his patient, mentioned in Dr. Hall's sensible publication, is guilty of gross dereliction of duty, and deserves not the encouragement of the public.

Hildenbrand, in his *Institutiones Clinicæ,* has laid down a series of concise precepts for examining a patient and investigating the nature of a disease, which many of our readers would do well to consult. We presume that Dr. Hall is not unacquainted with the work in question.

Hildenbrand insists much on the length and minuteness of a first examination: on it, says he, depends chiefly the confidence which a patient will place in his medical attendant. It is, therefore, highly necessary to conduct such examination with every requisite degree of care and accuracy.

The principal objects to which a physician, on his first visit to the bed-side of a patient, should direct his attention, are—1, the condition of the patient, his disposition and propensity to certain maladies; 2, the occasional, or accidental, causes of the complaint; and, 3, the progress and symptoms of the disease under consideration.

The first question naturally resolves itself into the following subdivisions:—1. Sex of the patient; 2, age; 3, temperament; 4, complexion; 5, mode of life; 6, occupation, profession; 7, hereditary constitution; 8, idiosyncrasy; 9, miscellaneous observations; 10, deductions from the history of former morbid affections.

In investigating the second question, or that which relates to the accidental causes of the disease, we shall find great assistance from—1, the suggestions of the patient himself, whose inward feelings are seldom deceptive; 2d, from the answers we get to interrogations directed to the detection of the cause; and lastly, 3, from the physician's own personal observations, without the interference of the patient.

In pursuing the latter branch of investigation, it is necessary to have in view, A, the endemical diseases; B, the most prevailing character of certain diseases peculiar to the country in which the patient resides; C, the reigning epidemics; D, the season of the year and the atmospherical phenomena; and, E, the contagious disorders.

The third question to be attended to, as we have already observed, is that which relates to the progressive march of the disease, and its concomitant symptoms. To do this effectually, it is necessary that the physician should be made acquainted with the *anamnestic* stage of the disorder, or that stage of it which has elapsed previously to his first visit, and the history of which embraces, 1, the first period at which the disorder made its appearance; 2, the phenomena presented by it subsequently to its first invasion; 3, those which accompanied its progress; and, 4, the remedies hitherto employed. He should then direct his whole attention to the present stage of the disease, and endeavour to ascertain the real nature of it, through his acquaintance with the diagnostic signs which characterize each complaint.

It is very immaterial whether we adopt a system of investigation founded on an anatomical division, or one which has physiology for its basis. Dr. Hall has given preference to the latter, in imitation of Chaussier and some others. Hildenbrand, on the contrary, recommends the former; first, because it is not easy to forget any very important question, when the anatomical division of the body is assumed as a guide in our researches; and, secondly, because we, thereby, avoid troublesome and unnecessary repetitions.

---

ART. III.—*A Manual of the Climate and Diseases of Tropical Countries; in which a Practical View of the Statistical Pathology, and of the History and Treatment of the Diseases of those Countries, is attempted to be given: calculated chiefly as a Guide to the Young Medical Practitioner, on his first resorting to those Countries.* By COLIN CHISHOLM, M.D. F.R.S. &c. &c. &c. 8vo. pp. v. 236. Burgess and Hill, London. 1822.

WE do not recollect a period during which a greater dearth of new medical publications (we do not mean of good ones, for those are always scarce,) has prevailed than at the present moment in this country. We are positively driven to notice the work before us, for want of a better performance; and, as among the four or five other works (none of them of importance,) that have appeared within the last year, and which have not yet been noticed by us, Dr. Chisholm's volume is the least reprehensible, we select it, with some pleasure, for present consideration.

In doing this, however, we shall confine ourselves to a superficial analysis of its contents only; for it would be a waste of time to attempt to examine, with a critical eye, the merits and defects of a work which, on the whole, calls for the exercise of the least pleasing part of our magisterial authority.

Dr. Chisholm's volume is divided into three parts: the first of which relates to the statistical pathology of tropical climates, more especially the West Indies; the second, to the diseases of hot climates, more especially of the West Indies; and the third to the malignant pestilential fever, commonly, but improperly, called yellow-fever. We have copied the author's own words.

The first chapter opens with an account of the general structure and appearance of the West-India islands, and their climate. The author observes, that these islands may be divided into the high and low, respecting surface; and he adds, a little farther, that the distinguishing form of the summit, in all the mountainous islands, is the cone; a feature chiefly observed, he believes, in volcanic countries. We will admit this to be the fact, although we are aware of its not being, for instance, with respect to Curaçoa, Cuba, St. Domingo, &c.: how can it have a powerful influence on the atmosphere of the islands, their productions, and their salubrity, as Dr. Chisholm concludes?

Dr. Chisholm says, that the windings of the innumerable hills in these islands produce a change of temperature, which may be considered as the great agent of insalubrity. Is there, we ask, a single mountainous district in Europe, and particularly in the southern countries of it, to which the character of endless alternation of hot and cold temperatures does not equally apply, without, at the same time, exhibiting that degree of alledged insalubrity which is said to prevail in the Antilles.

But the fact is, that the islands of the western Archipelago are by no means insalubrious; and we, who have had occasion to see some hard service in those seas, and resided in some of the islands, have no hesitation in saying that, with one or two exceptions, it is difficult to find a more healthy climate in the Atlantic. One has heard a great deal, of late years, of *inter-tropical* diseases—and heaven knows what; and big books have been written on distempers of hot climates, by persons who have never been in them, or who have, at most, spent a few months in one particular climate south of the Cape, in a man-of-war, under circumstances by no means favourable to the acquisition of knowledge respecting the diseases which prevail on the vast continent they never visited, or among the numerous colonies scattered in the Indian seas, the names of which they learned from a mere inspection of the map. This species of charlatanery must now be exploded ; and the public should be told, that all the frightful descriptions contained in books of the *essential* and *inherent* insalubrity of hot climates, are bubbles of the brain. Were it necessary to quote any authority in support of this assertion, we should bring forward Dr. Chisholm himself, who, after having descanted on the unhealthiness of the West-India islands, suddenly breaks upon us with the following curious observations :

"That they [the islands] would prove so [very healthy] to every description of their inhabitants, European and native, British and French, were they all equally careful to avoid those excesses, and those imprudencies in diet and personal exposure, which in all climates are dangerous, and too often fatal, may be demonstrated in many instances. The uninterrupted health and longevity of the French and Creole inhabitants of both sexes, are the result of an active, prudent, and temperate life. Eighty, ninety, a hundred years, is by no means an uncommon age among these people: nor does this appear to be owing to their residence being higher and cooler than that of the British, although that, as a general cause of health, is, as I have already remarked, particularly remarkable; for many European French, and some Creoles, possessing fine plantations on the coast, enjoy the same exemption from disease."

Although Dr. Chisholm has committed the error of assimilating the meteorology of Demerary (with which he seems best acquainted,) with that of the West-India islands in general,—an error which is the most surprising, as the difference of climate between that colony and Cuba, for instance, is as great as between the east and west coast of Africa,—we consider his description of the climate of Demerary so interesting, that we shall offer no apology for extracting it entire.

"The year, in the West Indies, is divided into two portions, the dry and the wet seasons. Some divide each of these also into two, which they call the long and the short winter, and the long and the short summer. This subdivision is not always observable in the islands, but uniformly so on the continent (Demerary.) The dry season is the portion of the year between the beginning of December and the end of April. In its ordinary course, it is pleasant and healthy. It is almost constantly ushered in by northerly winds, which prevail, with little variation, the whole of its duration; but they are most chilly, dry, and boisterous in December, January, and February. The total suspension of vegetation, during this season, is surprising, and seems to be occasioned by the want of moisture and exiccative shriveling quality of the north winds. All deciduous trees are stripped of their leaves ; the pastures become parched and brown ; and the cane-fields assume the autumnal hue of northern climes. The latter months of this season are the most pleasant of the year: for nothing can exceed the freshness of the mornings, and the softness of the evenings, of April and May. The rainy season includes the two last months of summer, all autumn, and generally the first month of winter. Its approach is awful, and always indicated by thick fog resting on the tops of the higher mountains, soon followed by heavy, dark, watery clouds, slowly rolling along from the N.E. in terrific volumes, enveloping the mountains, and darting bright electric corruscations from their edges. From these clouds tremendous torrents of water suddenly fall, and, rolling down the bottoms of ravines and the beds of rivers, carry every thing before them, and discolour the sea several miles in every direction with ochry earth washed from the interior mountains. This portion of the year is not, however, composed of a continued series of rainy weather. Many successive days occur of dry weather, chiefly in August and September, and are distinguished by an almost insupportable sultriness and closeness. As the surface is mountainous, such as many of the islands,—or woody, as Demerary and the larger islands,—so is the quantity of rain which annually falls. Thus, at Martinico, 100 inches, on an average, fall; at Demerary, 80. During this season, southerly, westerly, and easterly winds blow, and are always hot and sultry. The months of March and September are particularly stormy, in all the islands; but the more northerly and southerly in latitude seldom experience the disastrous effects of those awful storms called hurricanes: those tremendous atmospheric whirls seem to observe a uniform limited tract, seldom exceeding the 18th degree north and the 11th of south latitude, and maintaining a diagonal line between these degrees. Thunder and lightning are not so frequent as the geographical situation and local circumstances of the islands might lead to expect. In the dry season they never occur. Southerly winds generally accompany them. The thermometer (Fahrenheit's) almost uniformly exhibits the following movements:—At 7 A.M. the mercury begins to rise, and continues to do so till 1 P.M.; from which time till 4 P.M. it is stationary: it then begins to fall, and continues to do so till about 10 P.M.; from which time till 7 A.M. it is again stationary. This routine of temperature is disturbed, only, when any remarkable change takes place in the atmosphere, such as much rain, attended with strong wind. The greatest change from this cause I have observed is 10°, the least 4°. The thermometer, exposed to the direct rays of the sun, has risen in ten minutes to 130°, or 42° above its stationary point at 1 P.M. of that day: 30° may, however, be considered the medium difference between the heat in the shade and in the sun. The medium difference between the heat of the atmosphere at one and ten P.M. is 9°.—I may here take notice of the difference of temperature produced in water by the state of the atmosphere during the day and night. It shows how extremely sensible the human body is to the smallest deviation from the usual heat it is exposed to, and that our sense of cold in this climate is merely relative. At 10 P.M. into a Spanish unglazed guglet, full of water, I plunged a small thermometer; in five minutes the mercury sunk 3°, its stationary point then, in the open air, being 82°. I then placed the guglet, with the same water, in an open window, where it was left till 6 A.M. On plunging the thermometer then into the water, the mercury sunk to

72°, or 10° lower than the stationary point during the night. This degree of coolness in the water rendered it, to my feeling, rather cold and chilling: —this I have frequently repeated. A cold bath, constantly covered from the sun's rays, may be generally considered about 70°; a degree of cold which, if inconsiderately applied to the body, by suddenly plunging into it, has often produced the most fatal consequences. The greatest height of the thermometer (Fahrenheit's) at 1 P.M. in the shade, is 92°; the least, 74°; the medium is, of course, 83°. In Demerary, differing essentially in all its local circumstances from the islands, and only 6° from the equator, the medium heat is only 80°. The barometer, within the tropics, is little affected; its greatest range not exceeding, in any year, one inch." (p. 4, 5.)

In chapter third, the author has detailed certain means of preparing the European constitution for what he, oddly enough, calls the process of assimilation to the tropical climates, or what the French, more appropriately, term "*pour s'acclimater.*"

"In crossing the Atlantic to the West Indies, the change of temperature is gradual; and it is not till the 24° of N. latitude is reached that the thermometer rises to a height varying from 72° to 80°, when the human body perceives itself materially affected by the heat. The difference between the heat in the shade and in the sun is, in this latitude, almost never more than from 6° to 8°.—At this period of the voyage, catarrhal complaints, and determinations to, and slight congestions in, the lungs, the liver, and head, together with eruptions on the surface and a tendency to stupor and drowziness, are felt. The presence of these, and the degree of their violence, depend altogether on the person's being accustomed to continued and uniform heat, as well as on his temperament at the time: consequently, youths and men in the prime of life, on their first voyage, are most acted on by the augmentation of heat. If nothing is now done, a febrile diathesis, of more or less danger, may be excited; but, if measures of common prudence are resorted to, the symptoms I have mentioned gradually disappear, the perspirative process readily carrying off the surplus of heat." (p. 11, 12.)

The process of assimilation Dr. Chisholm alludes to, is greatly facilitated by resorting to the following preparation:

"On reaching the northern tropic, or N. lat. 23°, every stranger to the torrid zone should be bled to an extent proportioned to his age and strength; and a pill of five grains of calomel given at night, and a saline purgative the following morning. The bleeding should be repeated, if necessary, once before landing; but the calomel and salts should be frequently resorted to, and this will be more necessary should there be a disposition to constipation. I have already observed that, on approaching the tropics, a considerably tendency to congestion is perceived: this greatly increases on a further advance, more especially hepatic congestion, which, in fact, is the most serious consequence to be apprehended on entering the tropics.—Nothing more effectually obviates this than moderate bleeding, and mercurial and saline purgatives. To assist this course, the diet should be made as cooling as possible. Perspiration being the great means employed by nature to carry off the superfluous heat, every thing which tends to restrain it should be avoided: dilution is therefore, in every respect, highly necessary; and it is evident that, with this view, water is the fluid best calculated, for, whilst it promotes perspiration, it necessarily prevents determinations and congestions. Should any addition be deemed necessary, it should be such as may render the water more pleasant, and give it a greater tendency to increase alvine evacuation and perspiration. These effects cannot be promoted by the copious commixture of ardent spirits, so freely indulged in by soldiers and sailors; nor can the intention of dilution be fulfilled by the large quantities of wine and fermented liquors indulged in to an equally destructive excess, by a great majority of men in the higher walks of life. When the foregoing course is begun, it should be further seconded by daily cold bathing, either by immersion or affusion. No rule can be more easily adopted and pursued on ship-board, so that any direction for carrying it into effect seems quite unnecessary." (p. 12, 13.)

In treating of the adventitious and sporadic diseases of the West Indies, Dr. Chisholm takes the opportunity of declaring his conviction that the malignant pestilential fever, vulgarly, but improperly, called yellow-fever, is due to imported infection. He then enters into the consideration of the means of prevention depending on public police, on those depending on individual precaution, and on the medical means of prevention. Among the latter, Dr. Chisholm has very properly enumerated the effusion of chlorine in infected places; the application of which, he informs us, was much resorted to, on Mr. Cruikshanks, chemist to the Board of Ordnance, discovering a simple mode of disengaging that gas.

"Let four pounds of common salt be intimately mixed with a pound and a half of manganese reduced to fine powder; introduce them into a leaden vessel with a cover, and add about two pounds of water, through a hole in the cover. By means of a glass funnel, introduce, by degrees, and at different times, three pounds and a half of sulphuric acid. On every addition of the sulphuric acid, the oxygenated muriatic gas (chlorine) will escape in great abundance through the spout, and may be directed to any place, and in any quantity, we please. By the application of a moderate heat to the vessel, a very considerable quantity of gas may be obtained from the same materials, after they have ceased to give out any in the cold. This gas may be employed in moderate quantities to correct putrid and offensive smells in any particular ward or chamber, without removing the patients." (p. 28.)

The diseases treated of in the second part of Dr. Chisholm's book are these:—Yellow remittent fever, intermittent fever, dysentery, hepatitis, enteritis, cholera morbus, cholera pictonum, chorea, worms, pleurisy, pneumonia, and phthisis pulmonalis; coup de soleil, phrenitis, and hydrocephalus; rheumatism, ophthalmia, and other local inflammations; ulcers, tetanus, hydrophobia, catalepsy, hysteria, &c.

Yellow remittent fever.—This is the product of exhalation from damp, unventilated places; and its symptoms agree almost with those of common typhoid fever. Dr. C. has detailed them with great minuteness.

*Dissection.*—The inspection of a great number of bodies, dead of the yellow remittent fever, gave a result almost always uniform.

"The principal organ affected was the liver; and it is not a little singular that, in those cases of the worst kind of this fever which terminated fatally, this viscus was found either in a loose, dissolved, putrid state; or sphacelated, and having the consistence, the feel, and colour of rotton cork; or full of abscesses:—in such cases, too, the biliary ducts were rendered impervious by stricture; little bile in the gall-bladder, and that always black, ropy, and granulated. In many cases, several portions of the intestinal canal were inflamed, particularly the duodenum; and here and there evident marks of gangrene were observed. The spleen was greatly enlarged. The mesenteric glands were, generally, enlarged, in a state of scirrhus, or full of pus. The stomach, in general, had its coats thickened, the villous coat abraded, and the blood-vessels much distended. No bile was found in it; but black mucus, or the fluid discharged, resembling coffee-grounds. Every part of the body appeared tinged with a deep-yellow colour.—It was remarkable that the blood in the large vessels was in very small quantity, and had more the appearance of serum or water, tinged with a yellowish-red colour, than of blood." (p. 44.)

*Treatment.*—Copious bleeding and plentiful alvine evacuations are recommended. Five grains of calomel, with or without opium, according to the state of the stomach and bowels, are to be given, and repeated every three or four hours. Dr. C. says, that thirty or forty grains have generally brought on salivation; after which, all alarming symptoms have disappeared, and the patient has become convalescent. Our personal experience is in direct opposition to this result of a practice, which has been since exploded, and which we are surprised to find Dr. Chisholm has again brought forward. Things have changed since this veteran practitioner tried his experiments on the military in the western hemisphere; and his pathology, as well as therapeia, are several years behind our modern practice.

Intermittent fever.—This fever is characterized by the same symptoms which distinguishes it from other fevers in colder climates. Dr. Chisholm also tells us, that the same remedies, bark and arsenic, prove successful in curing it. Here, then, the *intertropical* climate affects not the etiology of the disease.

Dysentery.—This complaint has been so ably described by

Mr. Bampfield, whose observations have been very judiciously copied and adopted by Dr. James Johnson, in his useful compilation on Diseases of Hot Climates, that we need not waste one moment of our readers' time upon it. Dr. Chisholm has not said the thousandth part of the good things Mr. Bampfield has written on this subject. He cannot, therefore, be referred to, or recommended as an authority in these matters.

There is a sort of a cock-and-a-bull story, in the shape of a pathological explanation of dysentery, from some late learned and ingenious friend of the medical staff of the army, in this part of the work, of which we are confident Dr. Chisholm himself must have grown ashamed, since he has had constant intercourse with the continental pathologist, whose sound views respecting morbid affections of the intestines have superseded all the scholastic rhapsodies of former times. The same observation applies to what Dr. Chisholm has advanced respecting chronic aphthæ present in dysenteric affections.

Hepatitis.—The doctrine of the hepatico-cutaneous sympathy has been so well and so properly laughed at, that we need not trouble ourselves with showing to Dr. Chisholm that he comes rather too late with it before the public, when he endeavours to account for the occurrence of this disease on gratuitous data, without one tittle of positive or experimental evidence. His chapter on Hepatitis is positively worse than bad; and we hesitate not in charging the author with following a practice in the treatment of it, which is "safest when not adopted."

But this is really an unprofitable occupation on our part, and we lack courage to go through the remainder of this book, with so little advantage to our readers, and no gratification to ourselves. Dr. Chisholm proceeds, much in the same strain, to give an account of the remaining diseases which he has been pleased to term diseases of hot climates; and it will be enough to say of it, that the difference between his doctrinal tenets and practical suggestions in almost all these complaints, and the more enlightened theory and practice of modern days, is so great, that his work cannot be looked into except as a curiosity, —as a rejected piece of antiquity. Dr. Chisholm's book ought to have been published forty years ago, and it might then have met with some *plauditores.*

From a very superficial perusal of the third part of this work, we believe we can say that it has stronger claims than the other two to commendation. We have, however, been so nearly teased to extinction with controversies about the contagious and non-contagious nature of the malignant pestilential fever, that we dread to approach the subject afresh, particularly when treated by a pen which has neither thrown the interest of novel views upon the question, nor the charms of elegant composition.

### DIVISION II.

### FOREIGN.

ART. III.—*Essais sur les Irritations Intermittentes; ou nouvelle Théorie des Maladies periodiques, Fièvres larvées, pernicieuses, et intermittentes en general, exposée suivant la Doctrine de* M. BROUSSAIS, *et appuyée d'un grand nombre d'Observations.* Par P. G. MONGELLAZ, D.M.P. 2 vols. 8vo. pp. 859. Paris, 1821.

It is long since the celebrated BACON, of Verulam, clearly demonstrated that the readiest way to forward the progress of science, was to make a particular abridgment of each, taken at different epochs, in order to show their gradual improvement, and to render their study easier. CASIMIR MEDICUS expresses himself in a similar manner, in the Preface to his Treatise on Periodical Diseases; and such were the motives which induced him to publish that book, which is still considered as a classical work, wherein we discover, amidst the most antiquated humoral theories, many valuable physiological observations. According to him, paroxysmal fevers, and periodical diseases without febrile symptoms, have a great affinity between each other: they separately depend on an identical internal cause, and are cured by the same treatment. This internal cause is the increased irritability of the primæ viæ, the abundance and the alterations of the bile and pituitous matter, indigestions, and worms. The causes of most periodical diseases originate in the lower abdomen, the stomach, and the intestinal canal. With regard to the treatment, he proposes, 1st, to expel all the extraneous and irritating contents of the stomach and intestines, by emetics, cathartics, clysters, frictions of laxative substances on the abdomen, and vermifugous medicines; 2dly, to diminish the increased irritability of the parts, and at the same time restore them to their natural tone, by bark, alone or combined with laxatives, vegetable acids or bitters, by narcotics, stomachics, lime, or orange-peel, by pepper in grains, (recommended by DIOSCORIDES, and recently proposed by Dr. FRANK,) and by exercise; 3dly, in cases of metastasis, by blood-letting, scarifications, and the application of leeches, cupping-glasses, and blisters.

We think the reader will be pleased to have an opportunity of comparing the doctrines of Casimir Medicus with those of Dr. Mongellaz: these kind of approximations are of too great utility to be neglected. We have exposed the *ensemble* of the ideas of Casimir Medicus without any exclusion, that we may not be accused of having praised the dead at the expense of the living.

M. MONGELLAZ was desirous of embracing, in a general manner, every thing which concerns intermittent disorders pathologically,—to present a rapid view of every periodical and intermittent disease,—to place them in apposition, in order to show the relations existing between them. He commences with the history of those periodical diseases of which the seat is known, to arrive, with more surety, to a knowledge of those intermittent fevers whose history offer many blanks, and whose symptoms are vaguely demonstrated. His object has been to throw light on the theory, and to present, in a detailed manner, all the relative indications as to the treatment of a class of diseases, and of an order of fevers, from which arguments are incessantly produced in opposition to the new doctrine, and which appear to be the last refuge of its adversaries. To M. Mongellaz, every disease, every periodical fever, is an intermittent irritation. He defines that irritation as an increased organical morbid action, which becomes manifest, with more or less regularity, at certain periods, in any parts of the body, subject to certain modifications. Irritation is to him a term analogous to inflammation, with this difference, that the first has a more extended sense, since it comprises the second, and that there is no inflammation without irritation; whilst irritation often exists without inflammation. Lastly, all intermittent irritations indicate an increase of vitality. Irritation is never produced by weakness; even that which is apparent from pain only, is not occasioned by it. He pretends that each paroxysm is independent of the one preceding, and that it is not connected with that which succeeds it, by a cause inherent to the economy. He denies that dependency, because it has not been demonstrated,—because the parts exhibit no traces of irritation between the paroxysms,—and because the interval is often of considerable duration, such as ten, fifteen days, a month, a year, and more. Though these different reasons deserve little reflection, nevertheless we cannot forbear asking the author if there be no necessary connexion, no dependence, between the paroxysms of a tertian malignant fever, which in a few days occasions the death of a traveller imprudent enough to pass the night in the "campagna di Roma?" No doubt, he will allow that the first attack is the effect of the deleterious emanations to which this traveller has been exposed; but, in his theory, will he tell us why the disease did not there terminate; why a second, and why a third paroxysm should follow; if he denies that the emanations could have developed a cause inherent to the economy? It was necessary to attempt to discover the cause, not to explain its mode of action; but to know what it is, certainly it exists as well in intermittent as in continued diseases. The cause, which must not be sought for, because, most probably, it will never be discovered, is that of the intermittence; and not that of the phenomena which characterize the intermittent as well as the continued diseases.

The author divides the intermittent irritations in four kinds: the one have their seat in the capillary sanguineous system; he calls them inflammatory: the others attack the same system, but exhibit, in addition, a sanguineous effusion, more or less considerable: those are hemorrhagial irritations. Again, others affecting the lymphatic, the absorbing, excreting, and secreting systems, assume the name of sub-inflammatory, or lymphatic; and lastly comes the nervous irritation, so called from the affection of that part of the system. This division is more seducing than practicable, as will be seen by the difficulty which M. Mongallez himself experiences when he attempts to class the facts he adduces in support of his opinions. Nothing can demonstrate more clearly this uncertainty, than the characters which he assigns to each of these irritations: thus, redness indicates the presence of inflammation; the absence or non-predominance of that symptom distinguishes sub-inflammation, as well as an affection of the lymphatic system. As to the nervous, pain is often the only remarkable phenomenon; but it often happens, says he, that the sanguineous capillary vessels, intimately connected with the nerves, take part in the irritation, which at first was merely nervous, and soon an accumulation of liquids, and concomitant symptoms of inflammatory irritation, supervene: from which M. Mongellaz ought to have concluded, that nothing is more futile than the distinction of the nervous from the inflammatory irritation. All these, says he, may be combined, in some degree, in the same organs; yet he assures us, that inflammatory intermittent irritations, which primitively attack the sanguineous capillary system, do not communicate often with the white capillary vessels and concomitant nervous fibres. There is here a double error: firstly, it is precisely the combination of the irritation of the sanguineous capillary vessels with the lymphatics which constitute, according to the principles of physiological doctrines, those obstructions so common at the end of repeated paroxysms in intermittent fevers. Next, we know not what can be understood by an inflammatory irritation, which primitively attacks the sanguineous capillary system; but it is certain that the irritation transmits itself to the concomitant nervous fibres, whenever an inflammatory irritation is painful; which, by the bye, is no rare occurrence.

Amongst the general expressions which M. Mongellaz makes use of in the first chapter of his work, we consider the following proposition too generally applied: "The use of bark generally suffices to cure all kinds of intermittent irritation, and the efficaciousness of this medicine is equally great, whatever may be the nature, the seat, and the type, of the intermittence of the irritation." This proposition is too general; for who is unacquainted with the frequent failure of bark in cases of epilepsy, hysteria, chorea, mania, chronic rheumatism, periodical neurosis, and intermittent nausea occasioned by a chronic irritation of the stomach? Did bark save the patients affected by the fevers of Walcheren and Italy? He should merely have said, that bark generally succeeds in a great number of periodical diseases. Let us not be accused of minuteness because we insist on this point. General propositions remain alone in the minds of students, as well as with many practitioners; it is therefore highly important that they should never exceed the truth.

M. Mongellaz admits it as a principle, that any local irritation of moderate extent, either external or internal, may, by its sympathetic influence on the heart, stomach, &c. induce fever; in support of which, he quotes a case of quartan fever produced by the irritation excited by a "dens molaris," being upon the point of appearing, which Frank could not cure until the tooth had pierced the gum; and another case of intermittent fever, evidently owing to the excitement produced upon the mucous membrane of the urethra by a catheter, related by Gianini; from which we may conclude, that M. Mongellaz does not attribute all fevers to the irritation of the mucous membranes. We are glad to see that he is of our opinion on this point, as well as many other medical men; and, if we make this observation, it is merely to infer that we do not consider our opinion absurd, when we maintain that all fevers are not cases of gastro-enteritis. According to our author, scarcely any thing is found in the works of the antients tending to prove that they had any knowledge of periodical diseases, &c. This is not correct. Hippocrates, Galen, and most of the ancient physicians, have written on epilepsy and convulsions. M. Mongellaz, after having cited Mercado, whom he calls *Mercatus,* and Morton, ought to have added Casimir Medicus: he quotes him in another place, but he should not have forgotten him in this place.

The second chapter is dedicated to the external intermittent irritations; the third, to the internal ones, the malignant, and the ordinary intermittent fevers; the fourth and last to the inflammatory intermittent irritations,—that is to say, to the ordinary remittent and intermittent fevers, which generally have their seat in the organs of digestion. In this manner does the author pass successively in review, inflammation, hemorrhagies, sub-inflammations, and the external intermittent nervous fevers; next the internal ones; and lastly, the malignant and benign. Amongst the external intermittent irritations, the inflammatory are—ophthalmia, coryza, otitis, urticaria, scarlatina, erysipelas, phlegmon, rheumatismus, and podagra. The hemorrhagial comprehend hemorrhage from the umbilicus, epistaxis, and hemorrhagia buccalis. The sub-inflammatory are—anasarca, sweats, elephantiasis, dartrous eruptions, and swellings of the cervical glands, the inguinal, and the testes. The nervous are —cephalitis, hemicrania, and neuralgia sub-orbitaria; odontalgia, otalgia, and the nervous irritation of the inferior anterior part of the leg, and sciatica. Seventy-nine observations, taken from a great many different works, tend to prove that the intermittent type has been observed in all these diseases: nevertheless, on consideration, with regard to otitis and otalgia, the difference between them is not sufficiently great to justify the author in having so far removed them from the affections of the auditory organs. We can scarcely persuade ourselves that Barbette has seen an intermittent phlegmon. Can we, with M. Mongellaz, add, to examples of intermittent phlegmonous irritation, the following fact:—"M. Dupuy de Bellegarde relates an instance of a periodical inflammation of the throat, ending with the suppuration of an abscess, in which the patient felt no more of the disease till the following year, at the same epoch, when this affection returned, and terminated as before." Can we, conscientiously, on such examples, establish a new doctrine? There are too many observations of this kind in the work which we analyze. Here is one, which luckily occupies but a few lines:—"Schulze says, that he himself, being young, was subject, every month, to a bleeding of the nose, from the right nostril only."

Cephalalgia is certainly very often an intermittent irritation; but where is the proof of its being nervous? Is it because the patients complain of pain in their head? The redness of the tunica conjunctiva, and the increased secretion of tears, induces us to believe that this pretended neurosis is merely the symptom of a slight inflammation of the arachnoid, when it is not owing to gastric irritation.

The internal intermittent irritations are treated by M. Mongellaz, like those of which we have already spoken: it is absolutely the same plan continued. We do not consider it necessary to demonstrate that nothing is less physiological than a plan which removes apoplexia from catalepsia to associate it with croup; and which assigns no precise seat to epilepsia, mania, chorea, hysteria, &c. We will not attempt to discover whether asthma is an irritation; if the evacuation of semen, and the expectoration of pus be sub-inflammations; or whether convulsions and neurosis be synonymous. It is evident that M. Mongellaz, wishing to write the history of intermittent irritations, has only compiled that of intermittent symptoms, at least in a great part of his work. It is thus that intermittent gastritis is found disseminated amongst inflammations and sub-inflammations, under the names of cephalalgia, elephantiasis, &c. because they happen to be accompanied by pains of the head, or turgescence of the subcutaneous cellulary tissue.

Instead of confining himself to the collecting of observations on quintane, sextane, octane, decimal, mensual, sextimensual, and annual intermittent diseases, he should have assigned the seat, and indicated the nature, of the often continued and depraved habit from which the intermittent symptoms are derived, known under the name of asthma, cough, epilepsia, hysteria, &c.; and if the actual state of the profession does not allow him to pronounce formally on some analogous cases, he should, at least, have said as much to his readers.

M. Mongellaz quotes Hippocrates, Galen, Fernal, Baillon, Sylvius, Rivière, Vanhelmont, Sydenham, Bartholin, Ettmuler, Stahl, Hoffmann, Rega, Huxam, Fizes, Senac, Casimir Medicus, Baglivi, Grant, Cullen, Stoll, Tissot, Desbois de Rochefort, Grimaud, Pinel, and many others; who, says our author, all acknowledge the primæ viæ to be the seat of the cause of intermittent fevers.

Hitherto we have not said any thing concerning the treatment. Although the author has conducted that part of his subject with great care, his principles are those of the new doctrine, which are, already, so well known to our readers that it is unnecessary for us to dilate upon it in this already too long article; but I cannot omit to notice the solution which M. Mongellaz has given to the question, which terminates his work: "Does bark cure obstructions of the abdominal viscera?—that is to say, when we observe these organic alterations in a patient attacked by intermittent fever, is bark to be administered?" Before I hazard an answer, let us recal to mind that, in his opinion, gastro-enteritis is an ordinary intermittent fever; that obstructions are the effects of an irritation of the digestive organs; that the most renowned practitioners, and warmest advocates for bark, have not denied that this remedy might produce, or entertain, these obstructions; that, according to physiological doctrines, they are attributed to the untimely or abusive use of bark.—Who would believe, after all this, the answer which M. Mongellaz has brought forward?—"Yes, the methodical administration of bark is proper in all cases of intermittent fevers, though complicated with obstructions, dropsy, &c.: though these alterations may have happened at the end, or previous to the fever, (which last seldom happens,) we must administer bark," says he, "because we can never cure these obstructions without previously arresting the course of the fever. It is on account of the *trouble* which results from such a fever, and the shiverings which it determines, that the influence exerted on the obstructed organs increase their depraved condi[illegible]on." Let this physician explain to us, how a shiver can increase an obstruction; and why a speedy and methodical application of bark, by the most skilful medical men, repeatedly fails in numerous instances of tertian and quartan fevers? Till now we had thought, that the active stimulus of bark, not being counteracted by loss of blood, by an appropriate regimen, or considerable exercise, became hurtful, and favoured the formation of obstructions. We still maintain this opinion; because we consider it more consistent with physiology, and much more conformable to experience, than that of M. Mongellaz.

The facts which our author quotes are known to every medical man. The great question was not to prove the existence of intermittent irritation, but to demonstrate the propriety of considering as such malignant and benign intermittent fevers. To attain this object, it was sufficient to relate merely the general description which authors have given of these diseases, and to submit them to a physiological analysis: the work would have been shorter and better. The facts which M. Mongellaz advances are too numerous, if intended for students; and not sufficiently so for practitioners. A great number of them are exposed in too succinct a manner, too vaguely described,—to be considered as evidence in support of the opinions he proposes to develop. It is not by a painful assemblage of observations, many of which are defective, that we can expect to carry conviction to the minds of practitioners: it is by appealing to facts which they have themselves observed, by engaging them to follow, at least momentarily, the therapeutic method indicated to them: it is thus only that one can succeed, by degrees, in convincing others of the truth of any proposition.

Notwithstanding the critical remarks which we have thought necessary to make, and which, perhaps, we could have multiplied, this work deserves to be read. The author often shows himself a perfect master of his subject: the most experienced and successful practitioner could scarcely doubt the competence of this physician, whatever may be his youth. It is a singular method of refutation to say, "You write for the first time; *ergo*, you are wrong." Many interesting circumstances are developed by M. Mongellaz, concerning a doctrine which as yet has failed to unite the suffrages of all those physicians who do not consider it necessary to be guided by a mechanical instinct in the exercise of their profession.

